# Effect of Dinotefuran, Permethrin, and Pyriproxyfen (Vectra^®^ 3D) on the Foraging and Blood-Feeding Behaviors of *Aedes albopictus* Using Laboratory Rodent Model

**DOI:** 10.3390/insects11080507

**Published:** 2020-08-05

**Authors:** Younes Laidoudi, Djamel Tahir, Hacène Medkour, Marie Varloud, Oleg Mediannikov, Bernard Davoust

**Affiliations:** 1 AP-HM, Microbes, Evolution, Phylogeny and Infection (MEPHI), IHU Méditerranée Infection, IRD, Aix Marseille Univ, 19-21, Bd Jean Moulin, 13005 Marseille, France; younes.laidoudi@yahoo.com (Y.L.); djamel.tahir@yahoo.fr (D.T.); hacenevet1990@yahoo.fr (H.M.); olegusss1@gmail.com (O.M.); 2 IHU Méditerranée Infection, 19-21, Bd Jean Moulin, 13005 Marseille, France; 3 Ceva Santé Animale, 10, Av de la Ballastière, 33500 Libourne, France; marie.varloud@ceva.com

**Keywords:** *Aedes albopictus*, animal model, host selection, blood-meal identification, rodent-qPCR, Vectra^®^ 3D

## Abstract

**Simple Summary:**

Tiger mosquito (*Aedes albopictus*) is a harmful vector involved in the transmission of several diseases to humans and their pets. Currently, several veterinary products are used to prevent pets against bites of arthropod vectors. However, there is no available information on the effect of these products on feeding and host choice behaviors of *Aedes albopictus* in the presence of treated and untreated hosts, as is the case of treated dogs present in close physical contact with their owners. The present study investigated the effect of a spot-on product (Vectra^®^ 3D) on the feeding and host choice behaviours of *Aedes albopictus* when treated and untreated hosts are presents. Laboratory rodent model was performed to simulate the natural conditions. Rat and mouse hosts were alternately treated with Vectra^®^ 3D and exposed simultaneously to starved mosquitoes. Results showed that Vectra^®^ 3D-treated hosts are perfectly protected against up to 82% of mosquitoes. While up to 21% of mosquitoes were repelled from untreated hosts when these latter are present in close physical contact (30 cm) with treated ones suggesting an indirect protection that can allowed the protection of owners who treat their pets with Vectra^®^ 3D.

**Abstract:**

Dinotefuran-Permethrin-Pyriproxyfen (DPP) is used to kill and repel mosquitoes from dogs. However, the influence of the product on the host-seeking behavior of mosquitoes remains unknown. The interference of DPP with the host selection of unfed female *Aedes albopictus* was investigated. A total of 18 animals (9 mice and 9 rats) were divided into three groups of six animals each. DU: DPP treated rats (*n* = 3) with untreated mice (*n* = 3), UD: DPP treated mice (*n* = 3) with untreated rats (*n* = 3) and control UU: untreated mice (*n* = 3) and untreated rats (*n* = 3). In each group, the rats and mice were placed 30 cm apart. After sedation, the animals in each group were exposed twice (Day 1 and Day 7 post-treatment) for one hour to 71 ± 3 female mosquitoes. Mosquitoes were categorized after the 2-h post-exposure period as dead or alive. Blood-meal origin was determined from mosquitoes using a newly customized duplex qPCR. The highest values of forage ratio (1.36 ≥ *wi* ≤ 1.88) and selection index (0.63 ≥ *Bi* ≤ 0.94) for rat hosts indicates a preference of mosquitoes for this species as compared to mice when co-housed during the exposure. The mosquitoes only seldom fed on mice, even in the untreated group. The anti-feeding effect of DPP was therefore only assessed on rat’s hosts. The results showed that DPP, when directly applied on rats, provided a direct protection of 82% and 61% on Day 1 and Day 7, respectively, while when applied on mice hosts (UD), the DPP provided an indirect protection of 21% and 10% on Day 1 and Day 7, respectively. The results showed also that DPP, when applied on rats, provided a direct protection against *Ae. albopictus* bites. This effect did not result in increased exposure of the untreated host placed in the same cage at a distance of 30 cm.

## 1. Introduction

The Asian tiger mosquito, *Aedes albopictus* (Skuse, 1894), is considered the most invasive mosquito species in the world [1]. *Ae. albopictus* is a forest species that has adapted to rural, suburban, and urban environments In recent decades, this species of mosquito has spread throughout the world, from Asia to the Middle East, Australia, Africa, Europe, and Americas, especially through the international trade of used tires [2,3,4]. 

*Ae. albopictus* is a day biter and unusually aggressive mosquito, deteriorating life quality and may induce hypersensitivity reaction [5,6]. This mosquito has an opportunistic feeding behavior on a wide range of hosts, such as domestic and wild animals, reptiles, birds, and amphibians as well as humans [7,8], with a preference for mammalian host, especially humans, cats, and dogs [9]. This is why it has become a major pest in many communities.

*Ae. albopictus* is a competent vector of the main deadly viruses such as chikungunya, dengue, Zika, Japanese encephalitis, West Nile, yellow fever, and Usutu [10], and acts as a competent vector for filarial parasites, such as *Dirofilaria* spp. in Asia, North America, and Europe [11]. *D. immitis* induces canine and feline heartworm disease (Syn. cardiopulmonary dirofilariasis) and human pulmonary dirofilariasis, while *D. repens* causes respectively subcutaneous and subcutaneous/ocular dirofilariosis in canine and human hosts [12].

Currently, the use of insecticidal repellents is the most effective measure to protect pets against hematophagous arthropods such as mosquitoes [13]. Among veterinarian products, Vectra^®^ 3D (Ceva Santé animale SA, Libourne, France), a broad spectrum topical solution of dinotefuran, permethrin, and pyriproxyfen (DPP), has been experimentally proven to be effective in blocking the feed of *Aedes* mosquitoes on dogs [14,15] and on mice [16]. It is noteworthy that rodent model remain the easier to set and less expensive than the other ones, such as canine, chickens, pigeons, and goats [16]. 

DPP compounds are contact poisons and do not require ingestion by the insect to be effective [17]. Dinotefuran and Permethrin act as synergetic compounds inducing continuous nerve stimulation, incoordination, tremors, and death of the insect [17,18]. While the third compound (Pyriproxyfen) acts as a suppressor of embryogenesis of the insect [18]. Despite the absence of spatial repellency, pet owners often report that they had less mosquito bites when they stay close to their treated dogs with DPP. Until now, experimental studies demonstrated the effectiveness of these products by using test treatment and control groups exposed one by one to the mosquitoes [19,20]. However, such demonstration may not depict the host-seeking and feeding behaviors of mosquitoes in the presence of both treated and untreated hosts as is the case in the real life. 

To determine whether the feeding behavior and host preference of mosquitoes (in this case *Ae. albopictus*) is influenced by the presence of a treated host with insecticidal repellent products, we conducted an experimental study in which two different rodent species were exposed simultaneously.

## 2. Materials and Methods

### 2.1. Laboratory Animals and Ethics Statement 

A total of eighteen healthy animals were involved in this study: (i) Nine adults (aged 6 weeks) male Sprague-Dawley rats with a mean ± standard deviation (SD) weight of 160.7 ± 3.6 g and (ii) nine adults (6-week-old), female Swiss CD1 mice weighing a mean ± standard deviation (SD) of 20.16 ± 0.4 g (Charles River Laboratories, Écully, France). The animals were housed in groups of three individuals per cage of 58 × 36 × 20 cm for rats and 50 × 20 × 20 cm for mice, at a temperature of 22 °C under an LD 12: 12 h cycle. They were fed an appropriate maintenance ration of commercial food pellets (SAFE D03 Rats Mice breeding diet, usine SAFE, Augy, France). Filtered tap water was available ad libitum. Animals were checked by the veterinarian once a day. They were handled according to French rules on the protection of animals used for scientific purposes (decree no. 2013-118; 1 February 2013, Paris) [21]. The experimental protocol was reviewed and approved by the Ethics Committee for Animal Experimentation at Aix-Marseille University (approval n° 201602071706710).

### 2.2. Treatment Administration

Dinotefuran, Pyriproxyfen and Permethrin (DPP) are the active ingredients of the insecticide Vectra^®^ 3D (Ceva Santé animale SA, Libourne, France) product and are concentrated at 54.00 mg/mL of dinotefuran (CAS number: 165252-70-0), 4.84 mg/mL of pyriproxyfen (CAS number: 95737-68-1) and 397.00 mg per mL and permethrin (CAS number: 52645-53-1). The minimal recommended dose of Vectra^®^ 3D is 0.12 mL/kg of body weight (BW) for dogs. This dose was adjusted for use on laboratory animals as described in anterior studies [22,23,24]. Briefly, the equivalent dose (Ed) for each animal unit was appropriately calculated as follow: the Ed expressed in mg/kg BW was obtained by multiplying the dog dose expressed in mg/kg BW by the appropriate conversion factor for each host, wherein the conversion factors reported in Reference [22]. We therefore transformed each dose from mg/kg BW to mg/m^2^ body surface area (BSA) by multiplying the dose by the correction factor (*K_m_*) as described in Reference [23]. Finally, the appropriate amount of each compound was obtained by multiplying by the BSA of each host as shown in Table 1.

After an acclimation period, the animals were allocated into three groups of three rats and three mice each and were named TU, UT, and UU according to their treatment statues, where TU corresponds to DPP treated rats (*n* = 3) with untreated mice (*n* = 3), UT: untreated rats (*n* = 3) with DPP treated mice (*n* = 3) and UU: untreated rats (*n* = 3) and untreated mice (*n* = 3). This latter was used as control group. Once treatment has been applied, rats and mice from each group were housed in separate cages and maintained under the same conditions. The exposure to mosquito bites was performed twice at day 1 and day 7 post-treatment in order to determine the persistence of the repellent and killing effects.

### 2.3. Source of Ae. albopictus 

The Marseille strain of *Ae. albopictus* mosquitoes was maintained in the insectary of our laboratory (Aix-Marseille University, Marseille, France) from 2012, without exposure to any insecticides. Eggs were obtained from adult females fed on defibrinated human blood (according to an agreement with the Etablissement Français du Sang) using a Hemotek (Discovery Workshops, Accrington, UK). Adult mosquitoes were maintained in a climate-controlled chamber (Sanyo incubator MIR-254-PE; Sanyo Electric Co. Ltd, Tokyo, Japan) adjusted at 27 ± 5 °C and 80 ± 15% of the relative humidity (RH). They were allowed to feed on a 10% sucrose-water solution. Adult females aged 4 to 5 days were starved for 24 h prior to each challenge exposure.

### 2.4. Animal Exposure to Ae. albopictus 

**Figure 1:**
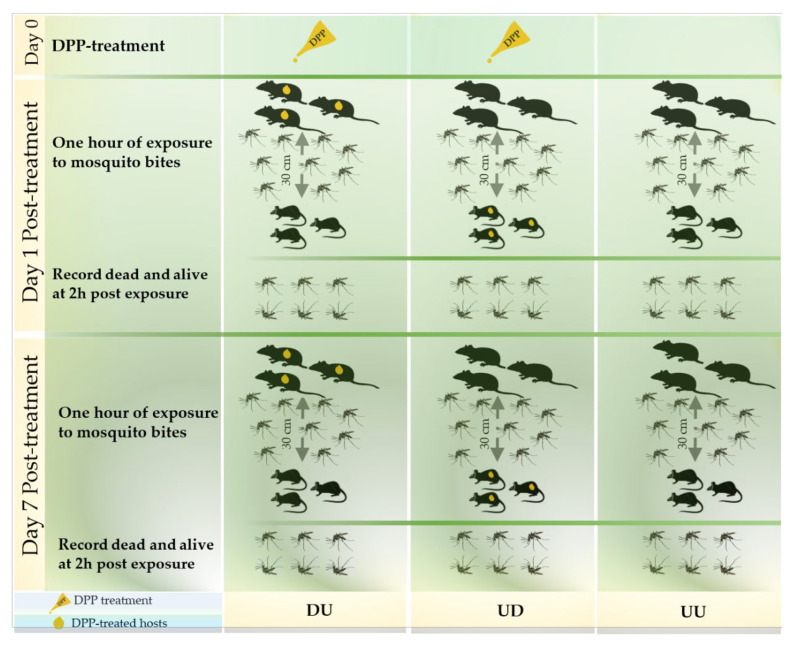
Overview of the study designs of the rat–mouse model.

At each exposure time, 71 (margin of error “me” = 3) adult females of *Ae. albopictus* mosquitoes were introduced in each of the 9 cages (W24.5 × D24.5 × H63.0 cm). After sedation by intraperitoneal injection of a combination of 90mg/kg BW ketamine (Imalgene^®^ 500; Boehringher Ingelheim SA, Lyon, France) and 10 mg/kg BW xylazine (Rompun^®^ 2%; Bayer Santé animale SA, Loos, France), the animals in each group were divided into three exposure subgroups. Each exposure subgroup was made of one rat and one mouse and was exposed for 1 h to mosquitoes on Day 1 and 7 post-treatment. A distance of 30 cm separated the sedated rats and mice in the exposure cages (Figure 1). The animals were carefully observed during and after the exposure to mosquito bites. The evaluation gird (Table S1) was adapted from the Reference [25] and was used to evaluate the tolerance degrees of the experiment by the animals in order to avoid any pain, distress, or discomfort throughout the whole experiment according to their signs. At the end of the exposure period, all animals were carefully reintroduced in their appropriate cages and surveilled until they woke up. Mosquitoes were then examined and classified as dead or alive at two hours after the exposure period. The engorgement determination was confirmed later by qPCR.

### 2.5. Engorgement Determination and Blood-Meal Identification

We developed a duplex qPCR to detect and discriminate between mosquito blood-meals taken from rat and mouse. The gene encoding for the adrenergic receptor subtypes alpha 2b (ADRA2B) was selected for the development of a discriminatory duplex-qPCR. The ADRA2B is known to discriminate between rodent species [26,27,28,29]. 

The available sequences of the ADRA2B genes of *Mus musculus* (mouse) and *Rattus norvegicus* (rat) were retrieved from GenBank database, then aligned using ClustalW application with BioEdit v 7.0.5.3 software [30]. Designed primers (Fwd-mu-rat-1117: 5′-GAGATCCTCAGGTGGGGTG-3′ and Rwd-mu-rat-1397: 5′-AACCAGGAGGTAGAGACACC-3′) target a partial sequence (280 bp) of ADRA2B gene of both rat and mice. The specificity of the qPCR was confined to the TAMRA probes, namely: P-mu-1343: 6VIC-5′-TTACGAACCCTCATGGCGAT-3′ specific to *M. musculus* and P-rat-1354: 6FAM-5′- CACAGTGATATGGCTGTGCA-3′ specific to *R. norvegicus*. The assay specificity was validated using the single and mixed DNA of rat and mouse as well as a wide range of negative controls from hosts (e.g., human, rabbit, cat, dog, donkey) and microorganisms (e.g., *Wolbachia* spp. *Leishmania*, *Dirofilaria* spp.) as previously described [31]. Once validated, the duplex qPCR was used to detect and identify blood-meals from all *Ae. albopictus* mosquitoes used in this study. Briefly, the DNA was extracted individually from every *Ae. albopictus* mosquito used in this study using the QIAGEN DNA tissues extraction kit (QIAGEN, Hilden, Germany), following the manufacturer’s recommendations. The qPCR reaction was carried out in a total volume of 20 µL, containing 3 µL of ultra-purified water DNAse-RNAse free, 5 µL of DNA template, 10 µL of Master Mix Roche (Eurogentec), 0.75 µL of each primer (at 50 µM concentration), and 0.5 µL of both UDG and each probe (at 20 µM concentration). The TaqMan qPCR run included two hold steps at 50 °C for 2 min followed by 15 min at 95 °C, and 39 cycles of two steps each (95 °C for 30 s and 60 °C for 30 s). qPCR reaction was performed in a thermal cycler CFX96 Touch detection system (Bio-Rad, Marnes-la-Coquette, France). Mixed DNA extracted from mice and rat bloods was used as positive control, while DNA extracted from unfed *Ae. albopictus* was used as negative control.

### 2.6. Data Analysis

For each exposure time, the Kruskal Wallis test with an exact computation of the *p-*value was used to compare between groups in terms of engorged, dead, and alive mosquitoes, as well as the blood-meal origin (rats, mice, or mixed hosts). The Conover-Iman test was used for pairwise comparisons between groups for each parameter. The difference was considered at *p-*value ≤ 0.05. The statistical analysis was performed using Addinsoft 2018 (XLSTAT 2018: Data Analysis and Statistical Solution for Microsoft Excel, Paris, France). 

The total number of alive *Ae. albopictus* females was transformed to the natural logarithm of (count + 1) for calculation of geometric means. The Abbott’s formula (Abbott, 1987) was applied to calculate the insecticidal effect induced by DPP treatment at each time of exposure: $\mathrm{Insecticidal}\;\mathrm{efficacy}\;\left(\%\right)=100\times \frac{NCi\;-\;\mathrm{NTi}}{NCi}$. Where NCi and NTi represent the geometric mean (GM) of alive *Ae. albopictus* in the control and treated groups, respectively.

The total number of engorged mosquitoes identified by qPCR for each group at each exposure time was transformed to the natural logarithm of (count + 1) for calculating geometric means (GM). The GM numbers of mixed (rat + mice) blood-meals were added to the GM number of single blood-meals upon each host. Two repellent effects were evaluated in each treated group at each time exposure: (i) the direct repellency against *Ae. albopictus* toward DPP-treated hosts (rats of DU and mice of UD) in comparison to the same hosts from UU, and (ii) the indirect repellency effect against *Ae. albopictus* toward DPP-untreated hosts (mice of DU and rats of UD) comparatively to the same hosts from UU. The direct and indirect repellency effects of DPP in treated groups (DU and UD) were calculated using Abbott’s formula (Abbott, 1987) [32] as follows: Anti feeding efficacy for host *i* (%) = 100 × (NCei−NTei)/NCe_i_. Where NCe_i_ and NTe_i_ represent respectively the geometric mean (GM) numbers of engorged females of *Ae. albopictus* upon host *i* from the control and treated group according to the qPCR results.

In addition, the forage ratio (*wi*) [33,34,35] and selection index (*Bi*) of Manly et al. [36] were calculated to assess feeding preferences of *Ae. albopictus* toward rats and mouse hosts regarding their treatment (DPP) statues for each exposure time. The percent of feeding upon host *i* was first calculated as the rate of engorgement upon host *i* (*Ri*) (i.e., considering the total number of females of *Ae. albopictus* used) using the following formula: $\%Ri=\frac{\left(Fei\;+\;Fex\right)}{\left(N+Fex\right)}$, where *Fei* is the total number of blood taken upon host *i*, *Fex* is the total number of mixed blood-meals, and *N* represents the total number of females of *Ae. albopictus* used in each group. In addition, in order to evaluate the feeding preference of *Ae. albopictus* toward hosts from each group, the rate of engorgement upon host *i* (*Oi*) was calculated by considering only the number of blood-meals taken by *Ae. albopictus* in each group, using the following formula: $\%Oi\;=\;\frac{\left(Fei\;+\;Fex\right)}{\sum_{i}^{2}Fe}$. Where, *Fei* is the total number of blood taken upon host *i*, *Fex* is the total number of mixed blood-meals and Fe is the total number of blood-meals. 

The forage ratio *wi* of *Ae. albopictus* for host *i* was calculated as: $wi\;=\;\frac{Oi}{Pi}$. Where *wi* is the forage ratio of *Ae. albopictus* for host *i*, *Oi* is the proportion of blood-meals upon host *i* and *Pi* is the proportion of host *i* available in each group. It was 50% at each time point. 

The selection index of *Ae. albopictus* for hosts was calculated as follows: $Bi\;=\;\frac{wi}{\sum_{i=1}^{n}wi}$. Where *Bi* is the selection index of *Ae. albopictus* for host *i*, *wi* is the forage ratio of *Ae. albopictus* for host *i* and *n* is the number of available blood-meals. Two types (*n* = 2) of hosts were available at each time exposure, values of 0.5 (1/*n*) of the selection index (*Bi*) indicate no preference, those below 0.5 indicate relative avoidance, and values above 0.5 indicate relative preference of host species *i*.

## 3. Results

The animals tolerated the treatment well for the duration of the experiment. Mosquitoes began attacking the animals immediately after they were released. Visual observations indicated that in the control group, mosquitoes preferred to bite rats over mice and seldom fed on the latter. This observation was confirmed following the molecular identification of blood-meals of mosquitoes. The comparative analysis between groups are shown in Table 2. At Day 1, the DPP treatment decreased the engorgement of *Ae. albopictus* mosquitoes in both DU and UD compared to UU, wherein the number of engorged mosquitoes upon treated hosts was significantly decreased in comparison to untreated ones. At Day 7, the same effect was only observed in DU. DPP treatment significantly increased the mortality of *Ae. albopictus* mosquitoes in both DU and UD compared to UU at each exposure time (Day 1 and Day 7) and was particularly high when the treatment was applied on rats (DU).

### 3.1. Insecticidal Efficacy 

A large majority of mosquitoes survived after exposure in the untreated group. In contrast, a significant mortality rate was recorded at each challenge time point within DU and UD in comparison to UU (Table 2 and Table 3). The insecticidal effect of DPP was 75.9% (Day 1) and 33.0% (Day 7) in Group 1 and 14.3% (Day 1) and 2.0% (Day 7) in Group 2 (Table 3).

### 3.2. Direct and Indirect Anti-Feeding Efficacy

The engorgement rate in the control group was 46.4% and 50.5% at Day 1 and Day 7, respectively. A significant reduction in the number of engorged *Ae. albopictus* females was observed in DU compared to UD and UU at each challenge time point, while no significant reduction was observed between UD and UU (Table 2 and Table 4). In all groups, the blood-meals taken upon rat hosts were always higher than those taken upon mice. In contrast to rat-blood-meals from the control group, where they ranged between 41.2 and 43.5, the rate of mouse blood-meals in the control group (Table 4) did not reach the threshold of 25% set by the WHO for the calculation of the repellent efficacy [37].

DPP repellent effects were calculated only for rat hosts (Table 4). The direct repellency was calculated from DPP-treated rats (DU) and it was 81.9% and 61.0% at Day 1 and Day 7, respectively. While the indirect repellency was calculated from UD, wherein the untreated rats were exposed simultaneously with DPP-treated mice. The indirect repellency was 21.1 and 9.9% at Day 1 and Day 7, respectively.

### 3.3. Host Preference 

**Figure 2:**
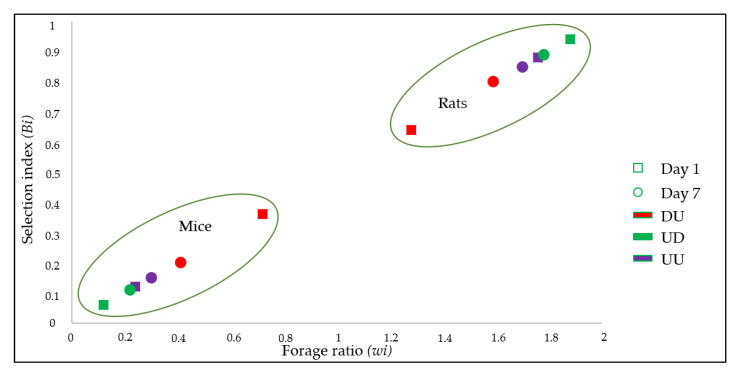
Scatter chart showing feeding preferences of *Ae. albopictus* mosquitoes in term of hosts and treatments according to the forage ratio (*wi*) and selection index (*Bi*). DU: DPP-treated rats vs. untreated mice, UD: DPP-treated mice vs. untreated rats, UU: Control group. Day 1 and 7: Time of post-treatment exposure.

Results of the forage ratio (*wi*) and the selection indexes (*Bi*) for each host are shown in Table 5 and Figure 2. Both *wi* and *Bi* indicated that rats were the preferred source of blood-feeding relative to mice in each challenge exposure irrespective to their treatment (Table 5, Figure 2). Data from UU indicates that *Ae. albopictus* had a tropism of 6–7 times higher for rat than for mice hosts.

## 4. Discussion

The present study focused on how the treatment by DPP influences the behavioral responses (blood-feeding and host choice) of *Ae. albopictus* mosquitoes toward both treated and untreated hosts available at the same time. The animal model was performed in order to reduce the potential confounders (e.g., rearing conditions of both animals and mosquitoes, treatment doses, experiment procedures, and the host defensive behaviors against mosquitoes) that can impair the results [38]. As expected, our data from the control group (UU) indicated that both the engorgement (mean = 48.5%) and the mortality rates (mean = 5.0%) of mosquitoes are in compliance with those recommended by the WHO, where the minimum (25%) and the maximum (20%) thresholds of, respectively, the engorgement and the mortality rates are necessary to validate the experiment [37,38]. 

As expected, DPP treatment induced a significant mortality of *Ae. albopictus* mosquitoes in both DU and UD compared to UU. The insecticidal effect was higher in DU (33–75.9%) than in UD (14.3–2%), thus highlighting the correlation between the insecticidal effect and the amount of DDP. The total amount of DPP used for the treatment of rats in DU was 5 times greater than that used to treat mice in UD. However, the insecticidal effect decreased rapidly (Table 1). Our data showed that the insecticidal effect had a rapid decrease from 75.9 to 33% and from 14.3 to 2% in DU and UD, respectively, at 7 days post-treatment. These results contrast with the previous reports where the single administration of DPP maintained a high insecticidal effect against mosquitoes for 30 days post-treatment [16,39,40]. The availability of untreated hosts at the same time with treated ones could limit contact between the mosquitoes and the DPP-treated hosts. DPP formulation was designed to repel and kill mosquitoes and other arthropods once the contact between the arthropod and the treated hosts is established [16,39,40]. 

Surprisingly, our data demonstrated that, despite the aggressive biting behavior of *Ae. albopictus* [40], the rate of blood-meals taken upon mice was lower than that taken on rats in UU and that reported by Tahir et al., 2017, using the same *Ae. albopictus* strain on mouse model [16]. This could be the results of body weight differences between rats and mice. The body size is positively correlated to the mosquito attractiveness [41]. Another hypothesis could be a change in trophic preference of *Ae. albopictus* when the simultaneous availability of both hosts given the opportunity to choose between them according to the feeding preference of the mosquito. Under natural conditions, *Ae. albopictus* preferred human blood-meals rather than the other vertebrates (e.g., chicken, dog, and calf) if the choice is offered [9,42]. Further studies are needed to elucidate these transient trophic behaviors of *Ae. albopictus* that can be affected in experimental as in natural conditions by availability and abundance of hosts [9].

In the present study, we demonstrated that the use of DPP treatment did not affect the feeding preference of *Ae. albopictus*, where blood-meals were primarily taken upon rats in all groups, regardless of the treatment statutes (Table 5, Figure 2). A few data are currently available on the feeding behavioral changes that might be induced by the insecticidal and repellent products [43]. A previous study demonstrated that despite the strong excito-repellency of pyrethroids, there was no diversion of host-seeking mosquitoes from treated cattle to nearby humans [44,45]. 

Another surprising finding in this study was the additional protection offered to untreated hosts when present near DPP-treated ones. This statement corroborates with the previous studies on malaria, where the community with pyrethroid-treated cattle showed a strong reduction in malaria transmission [44,45]. This is the result of the strong repellency induced by these pyrethroids [46,47]. Once contact is established between mosquito and repellent product-treated area [48], intense disorder of the mosquito’s sensory functions occurs, making it unable to detect the host and causing the mosquito to move in a disorganized manner away from a host to which it would otherwise be attracted [43].

In natural conditions, *Ae. albopictus* is one of the few is one of the few, if not the only mosquito species that showed the ability to feed upon rodent hosts such as Gray squirrel (*Sciurus carolinensis*) and White-footed mouse (*Peromyscus leucopus*) with a weaker engorgement rat compared to the other mammalian hosts [49]. However, the combined rat–mouse model used in the present study to assess host feeding patterns of *Ae albopictus* regarding to the type of hosts as well as the effect of the insecticide repellent (Vectra^®^ 3D) are limited by the absence of robust data on the feeding behaviors of *Ae. albopictus* towards rodent hosts. The hypothesis that rodents constitute a suitable host for this mosquito species cannot be ruled in the absence of further data. 

## 5. Conclusions

*Ae. albopictus* mosquitoes are very selective in terms of feeding preference when the opportunity of host choice is offered. DPP treatment does not alters the feeding behavior of *Ae. albopictus* and provides a partial protection to hosts in close physical proximity to treated animals. We encourage the use of this product on dogs to protect them and partially their owners against *Ae. albopictus* borne diseases such as dirofilariosis. However, further studies are needed to reinforce the usefulness of the combined models to simulate the real-life conditions that can occur between treated dogs and untreated owners.

## Figures and Tables

**Table 1:** Dose calculation of Vectra^®^ 3D applied on laboratory animals.

Species Description	Active Product	Conversion Factor from Dogs (dog dose mg/kg BW)	Dose mg/kg BW	Correction Factor (km)	Dose mg/m^2^ BSA	Dose Per Animal, mg	Vectra^®^ 3D (mL/animal unit)
Mouse (0.02 kg BW, 0.0066 m^2^ BSA)	Dinotefuran	6 (6.4)	38.4	3	115.2	0.76	0.014
	Pyriproxyfen	6 (0.6)	3.6	3	10.8	0.071	
	Permethrin	6 (46.6)	279.6	3	838.8	5.54	
Rat (0.15 kg BW, 0.025 m^2^ BSA)	Dinotefuran	4 (6.4)	25.6	6	153.6	3.84	0.070
	Pyriproxyfen	4 (0.6)	2.4	6	14.4	0.36	
	Permethrin	4 (46.6)	186.4	6	1118.4	27.96	

**Table 2:** Statistical analysis of engorgement status, blood-meal origin, and mosquito viability between the three groups.

		Molecular Identification of Blood-Meals				Viability at 2 h after Exposure		Total Used Per Group
	Statistics	Feed on Rats	Feed on Mice	Feed on Both	Engorged	Dead	Alive	
**Day 1**	DU	15 (4.9)	8 (2.3)	1 (0.6)	24 (7.9)	162 (53.6)	53 (16.1)	215 (71.6)
	UD	74 (21.9)	2 (0.6)	3 (0.8)	79 (23.0)	41 (13.5)	172 (57.3)	213 (71.0)
	UU	85 (28.2)	10 (3.2)	2 (0.6)	97 (32.1)	8 (2.4)	201 (66.9)	209 (96.5)
	**Kruskal Wallis test**							
	***p*-value**	**0.049**	0.997	0.996	**0.043**	**0.004**	**0.004**	/
	**Pairwise comparisons (*p*-value)**							
	DU vs. UD	**0.015**	0.079	0.674	**0.025**	**0.010**	**0.009**	/
	DU vs. UU	**0.027**	0.430	1.000	**0.014**	**<0.0001**	**<0.0001**	/
	UD vs. UU	0.657	**0.025**	0.674	0.653	**0.010**	**0.009**	/
**Day 7**	DU	33 (10.9)	7 (2.1)	2 (0.6)	42 (13.7)	96 (31.3)	129 (42.6)	225 (75.0)
	UD	78 (26.0)	8 (2.4)	2 (0.6)	88 (29.3)	31 (10.0)	187 (62.3)	218 (72.6)
	UU	87 (28.6)	13 (3.9)	3 (0.8)	103 (33.6)	13 (4.2)	191 (63.6)	204 (68.0)
	**Kruskal Wallis test**							
	***p*-value**	**0.039**	0.561	0.996	**0.05**	**0.004**	**0.029**	/
	**Pairwise comparisons (*p*-value)**							
	DU vs. UD	**0.028**	0.833	1.000	**0.030**	**0.010**	**0.028**	/
	DU vs. UU	**0.012**	0.314	0.674	**0.012**	**<0.0001**	**0.012**	/
	UD vs. UU	0.500	0.413	0.674	0.506	**0.010**	0.50	/

Values in bold are significant at the level alpha = 0.05; DU: Dinotefuran-Pyriproxyfen-Permethrin (DPP) treated rats and untreated mice, UD: DPP treated mice and untreated rats, UU: untreated rats and mice.

**Table 3:** Insecticidal efficacy of Vectra^®^ 3D against *Ae. albopictus* in combined mouse–rat model at 2 h post-exposure.

Groups/Times		Mortality Statutes of *Ae. albopictus*		Insecticidal Effect (%)
		GM	Percentage	
**Day 1**	DU	53.6	74.9	75.9
	UD	13.5	19.1	14.3
	UU	2.4	3.5	/
**Day 7**	DU	31.3	41.7	33.0
	UD	10.0	13.8	2.0
	UU	4.2	6.2	/

**Table 4:** Results of the engorgement rates on rodents (rats and mice) and the direct and indirect repellent effect of Vectra^®^ 3D against *Ae. albopictus* in the presence of both treated and untreated hosts respectively, at a distance of 30 cm between each.

Groups/Times		Engorged *Ae. albopictus*		Percent of Blood-Meals (% *Ri*)		Repellent Effect (%)
		GM	Percentage	Rats	Mice	Rat
**Day 1**	DU	7.9	11.2	7.4	4.2	81.9 ^a^
	UD	23.0	37.1	35.7	2.3	21.1 ^b^
	UU	32.1	46.4	41.2	5.7	/
**Day 7**	DU	13.7	18.7	15.4	4.0	61.0 ^a^
	UD	29.3	40.4	36.4	4.6	9.9 ^b^
	UU	33.6	50.5	43.5	7.7	/

^a^ and ^b^ indicate respectively the direct and indirect repellencies.

**Table 5:** Feeding preference of *Ae. albopictus* in each group towards treated/untreated hosts expressed by forage ratio and selection index.

Groups		Percent of Blood-Meals (% *Oi*)		Forage Ratio for Host *i* (*wi*)		Selection Index for Host *i* (*Bi*)	
		Rat	Mouse	Rat	Mouse	Rat	Mouse
**Day 1**	DU	64.0	36.0	1.28	0.72	0.64	0.36
	UD	93.9	6.1	1.88	0.12	0.94	0.06
	UU	87.9	12.1	1.76	0.24	0.88	0.12
**Day 7**	DU	79.6	20.4	1.59	0.41	0.80	0.20
	UD	88.9	11.1	1.78	0.22	0.89	0.11
	UU	84.9	15.1	1.70	0.30	0.85	0.15

## Data Availability

The data supporting the conclusions of this article are included within the article.

## Supplementary Materials

The following are available online at https://www.mdpi.com/2075-4450/11/8/507/s1, Table S1: Relationship between signs and degree of pain, stress, and discomfort.
