# Supplementary material for: Effect of Dinotefuran, Permethrin, and Pyriproxyfen (Vectra® 3D) on the Foraging and Blood-Feeding Behaviors of Aedes albopictus Using Laboratory Rodent Model
Source: Insects. 2020 Aug 5;11(8):507. doi: 10.3390/insects11080507 (PMC7469159; doi:10.3390/insects11080507)
Supplement: Supplementary file 1 [file insects-11-00507-s001.pdf]

Supplementary Material

Table S1. Relationship between signs and degree of pain, stress and discomfort adapted from Morton and Griffiths [25]

Score	Variable	Record
	**Body Weight Changes**	
0	Normal	
1	<10 percent weight loss	
2	10-15 percent weight loss	
3	>20 percent weight loss	
	**Physical Appearance**	
0	Normal	
1	Lack of grooming	
2	Rough coat, nasal/ocular discharge	
3	Very rough coat, abnormal posture, enlarged pupils	
	**Measurable Clinical Signs**	
0	Normal	
1	Small changes of potential significance	
2	Temperature change of 1-2°C, cardiac and respiratory rates increased up to 30%.	
3	Temperature change of >2°C, cardiac and respiratory rates increased up to 50%, or markedly reduced	
	**Unprovoked Behaviour**	
0	Normal	
1	Minor changes	
2	Abnormal, reduced mobility, decreased alertness, inactive	
3	Unsolicited vocalizations, self-mutilation, either very restless or immobile	
	**Behavioral Responses to External Stimuli**	
0	Normal	
1	Minor depression/exaggeration of response	
2	Moderately abnormal responses	
3	Violent reactions, or comatose	

2. Possible interpretation of total scores from an overall assessment of an experimental animal

A. Score 0 to 4: Normal

B. Score 5 to 9: Monitor carefully, should consider the use of analgesics and sedatives

C. Score 10 to 14: Ample evidence of suffering, some form of relief must be seriously considered; should be under regular observation; seek expert advice; consider termination

D. Score 15 to 20: Relief should be given, unless the animal is comatose. Is it a worthwhile experimental animal because physiologically it is likely to be abnormal? There is ample evidence of severe pain. If likely to endure, terminate the experiment.
